# Aberrant localization of apoptosis protease activating factor-1 in lipid raft sub-domains of diffuse large B cell lymphomas

**DOI:** 10.18632/oncotarget.13336

**Published:** 2016-11-14

**Authors:** Jayshree L. Hirpara, Thomas Loh, Siok Bian Ng, Wee Joo Chng, Shazib Pervaiz

**Affiliations:** ^1^ Department of Physiology, Yong Loo Lin School of Medicine, National University of Singapore, Singapore; ^2^ Experimental Therapeutics Program, Cancer Science Institute, National University Healthcare System, Singapore; ^3^ Department of Otolaryngology, National University Healthcare System, Singapore; ^4^ Department of Pathology, National University Healthcare System, Singapore; ^5^ Cancer Science Institute, National University Healthcare System, Singapore; ^6^ NUS Graduate School for Integrative Sciences and Engineering, National University of Singapore, Singapore; ^7^ National University Cancer Institute, National University Healthcare System,; ^8^ School of Biomedical Sciences, Curtin University, Perth, Australia

**Keywords:** DLBCL, apoptosome, Apaf-1, lipid rafts, ROS, Autophagy

## Abstract

Resistance to chemotherapy remains a challenge in the clinical management of diffuse B cell lymphomas despite aggressive chemotherapy such as CHOP and monoclonal CD20. Here we provide evidence that the apoptosome adaptor protein, Apaf-1, is mislocalized in primary cells derived from patients with diffuse large B cell lymphomas (DLBCL). Whereas, the total expression of Apaf-1 did not change, its sub-cellular localization was significantly different in DLBCL, compared to T cell lymphomas as well as cells derived from reactive lymphadenopathy biopsies. As expected, Apaf-1 was detected in the cytosolic fractions of non-B cell lymphomas and non-cancerous tissues; however, in B cell derived lymphomas the protein was detected in membrane raft sub-domains rather than the cytosol. Disruption of lipid raft structures resulted in the redistribution of Apaf-1 to the cytosol and restored apoptosis sensitivity of DLBCL. Furthermore, we identified novel small molecule compounds that target DLBCL by promoting Apaf-1 release form lipid rafts via mechanisms that involve an increase in intracellular reactive oxygen species production. Taken together, our results implicate Apaf-1 mislocalization as a potential diagnostic and prognostic marker for DLBCL, and provide a novel therapeutic strategy for circumventing the drug refractory nature of this sub-class of B cell lymphoma.

## INTRODUCTION

Non-Hodgkin Lymphoma (NHL) is a diverse group of cancers affecting the lymphatic/hematopoietic system. There are at least 30 types of NHL, with diffuse large B-cell lymphoma (DLBCL) and Follicular lymphoma being the most common and aggressive types of NHL. With timely and appropriate chemotherapy, such as CHOP and monoclonal CD20 (bivatuzamab), over half of the DLBCL patients are cured [1]; however, chemoresistance remains a major challenge, which potentiates the need for an improved chemotherapeutic strategy in relapsed patients. The molecular mechanisms underlying the resistance of DLBCL to chemotherapy include overexpression of the anti-apoptotic proteins belonging to the Bcl-2 family and/or the IAP (inhibitor of apoptosis proteins) family, thereby resulting in dysregulation of the apoptotic execution machinery [1].

The apoptotic program is orchestrated by a cascade of events resulting in the activation of caspases, either through the ligation of the death receptor (extrinsic signaling) or by engaging the mitochondrial amplification pathway (intrinsic signaling). The intrinsic death pathway is the common mode of chemotherapy-induced execution and requires the permeabilization of the outer mitochondrial membrane and release of death amplification proteins such as cytochrome C, Smac and AIF [2]. The cytosolic translocation of cytochrome C is critical for the formation and activation of the apoptosome, a death-signaling complex downstream of the mitochondria [3, 4]. The assembly of the apoptosome is brought about by the recruitment of cytochrome C and pro-caspase 9 to the adaptor protein Apaf-1 (Apoptosis Protease Activating Factor 1) in the presence of dATP, resulting in the activation of caspase 9, which then drives the activation of the executioner caspase 3 [5, 6]. Hence, death inhibitory proteins such as Bcl-2 indirectly inhibit the assembly of the apoptosome by regulating mitochondrial outer membrane permeabilization, while others such as the IAPs (Inhibitor of Apoptosis Proteins) control apoptotic execution downstream of the apoptosome by targeting caspase 9 [3].

There is ample evidence to implicate defective apoptosome assembly in the resistance of cancer cells to drug-induced apoptosis [7]. To that end, a number of recent reports demonstrate a link between chemoresistance and the cellular levels of Apaf-1. For example, epigenetic regulation of *Apaf-1* via its promoter methylation is associated with apoptosis resistance in malignant melanoma cells [8] as well as in some leukemias [9, 10]; however, in another study, inactivation of Apaf-1 was not corroborated in malignant melanoma [11] Defective apoptosome formation through LOH-mediated repression of Apaf-1 has also been reported in glioblastoma [10] and *Apaf-1* hypermethylation has been seen in bladder cancer [12]. Furthermore, loss of Apaf-1 has been linked to tumor aggressiveness in cervical cancer [13]. Corroborating the association between an absence or downregulation of Apaf-1 expression and drug resistance and/or aggressiveness of cancer, we previously reported a hitherto undefined mechanism of defective apoptosome signaling in human B cell lymphoma cell lines by demonstrating the sequestration of Apaf-1 to the plasma membrane [14].

Here we set out to investigate the clinical relevance of Apaf-1 mislocalization in primary cells derived from patients with lymphomas, and to establish a correlation between Apaf-1 mislocalization and apoptosis sensitivity in an *ex vivo* setting. We report that Apaf-1 mislocalization to lipid raft fractions of the plasma membrane is associated with a significantly muted response to apoptosis stimuli in DLBCL and follicular lymphomas. Furthermore, we identified novel small molecules that restored chemosensitivity of B cell lymphomas by affecting the release of Apaf-1 to the cytosol through an increase in intracellular reactive oxygen species (ROS), thereby facilitating apoptosis execution. These data provide a novel mechanism of loss of apoptosome assembly and function and its association with apoptosis resistance in clinical B cell lymphomas, which could have potential implications for the design and development of novel therapeutic strategies against the aggressive and refractory variants of B cell lymphoma.

## RESULTS

### Primary cells from B cell lymphoma patients are resistant to conventional chemotherapeutic agents

**Figure 1:**
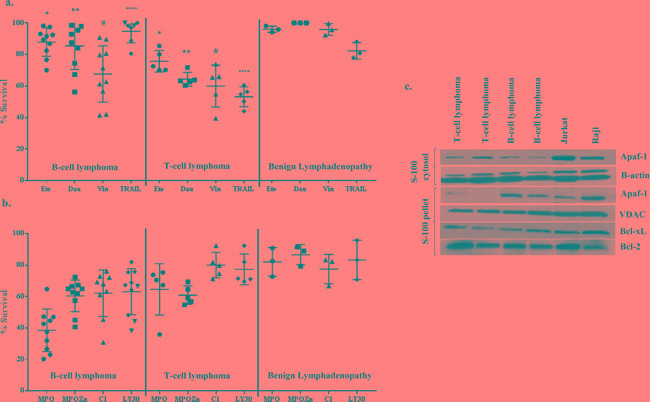
Primary cells from B cell lymphoma patients are resistant to conventional chemotherapeutic agents and contain significantly lower cytosolic Apaf-1 **a.**, **b.** 2×10^5^ primary lymphocytes/200μl/well in 96 well plates were exposed to Etoposide (5μM), Daunorubicin (0.4μg/ml), Vincristine (5μM), MPO (2.5μM), MPO-Zn (200nM), C1 (50μg/ml), LY30 (50μM) or TRAIL (100ng/ml) for 24 hrs. Cell viability was determined by the MTT assay. One way ANOVA multiple comparisons analysis was used for statistical significance (* *p* < 0.1, **p < 0.001, #*p* < 0.5, ****p < 0.0001) **c.** S-100 cytosolic and pellet fractions of primary cells from B-cell and T-cell lymphomas as well as from Raji and Jurkat cell lines were resolved by SDS-PAGE, transferred to PVDF and Apaf-1 was detected by immunoblotting using anti-Apaf-1. Membranes were probed with anti-β-actin and anti-VDAC-1 as loading controls for cytosolic and pellet fractions, respectively.

In order to understand the precise mechanism underlying the resistance of human B cell lymphomas to drug-induced apoptosis, we obtained biopsies from patients with a variety of lymphomas and performed magnetic separation of T and B cells as described in Materials and Methods. Primary cells were then subjected *ex vivo* to apoptotic stimuli, including the commonly used chemotherapeutic agents etoposide (1-10μM), daunorubicin (0.2-0.8μg/ml), vincristine (1-10μM), the death receptor ligand TRAIL (50-200ng/ml), as well as experimental small molecule compounds LY30 (25-50μM) [15], C1 (25-100μg/ml) [16, 17], MPO (1-10μM) [18], and MPO-Zn (100-400nM). As an internal control, two established cell lines, Raji and Jurkat, were used in parallel. Cell viability was assessed by the MTT assay following 24 h of incubation with the various compounds. Results show that primary cells from benign lymphomas or non-cancerous lymphoid hyperplasia were relatively insensitive to most drugs, while B cell lymphomas (BL Cells) in general were resistant to commonly used chemotherapeutic agents compared to T cell lymphomas (Figure 1a). This is in agreement with our earlier findings indicating B-cell lymphoma-derived cell lines were resistant to etoposide and other chemotherapeutic drugs. Interestingly, we provide evidence that primary BL cells were relatively more responsive to the experimental small molecules MPO[18], MPO-Zn (MPO analogue) and C1 [16, 17] (Figure 1b). We also tested the sensitivity of primary BL cells to the death receptor ligand TRAIL. TRAIL has been under clinical evaluation against because of its ability to selectively target cancer cells [19]. Firstly, primary BL cells, unlike TL cells, were not responsive to TRAIL, however pre-incubation of cells with the small molecule experimental compounds MPO, MPO-Zn, C1 or LY30 resulted in a significant amplification of TRAIL sensitivity (data not shown).

### Absence of Apaf-1 in the cytoplasm of primary BL cells

The aforementioned results provided evidence that clinical B cell lymphomas in general were relatively refractory to known chemotherapeutic agents, but did possess the functional armory to elicit an apoptotic response depending on the nature of the stimulus. Intrigued by these findings and based on our earlier report using established cell line [14], we set out to investigate the role of the apoptosome machinery in the resistance of primary BL cells to drug-induced apoptosis. Primary cells derived from 30 B-cell lymphoma patients, 7 T-cell lymphoma patients and 5 benign tumors were used for the assessment of Apaf-1 expression and its localization. Interestingly, Apaf-1 expression was detected in the S100 cytosolic fractions from primary TL cells as well as Jurkat cell line, however, the expression was virtually completely absent in the S100 cytosol but found mostly in the S100 pellet fractions of primary BL cells (Figure 1c, Supplementary Figure1a, b and c).

### Disruption of Apaf-1 from S-100 pellet increases caspase-3 activity

**Figure 2:**
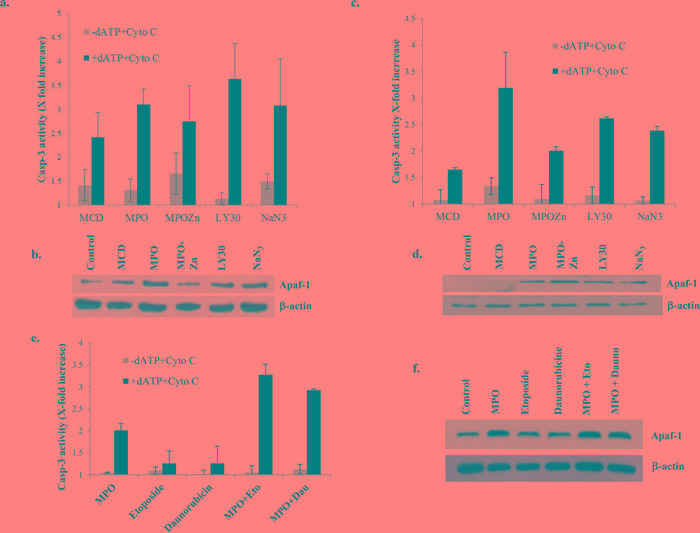
Disruption of Apaf-1 from S-100 pellet increases caspase-3 activity **a.**, **c.** S-100 cytosol was prepared from primary BL cells and Raji cells after incubation with MCD (200μM), MPO (5μM), MPO-Zn (200μM), LY30 (25μM) or NaN_3_ (5μg/ml) for 1hr. Caspase-3 enzyme activity was measured using DEVD-AFC in the treated and untreated S-100 cytosol of B-cell lymphoma in the presence and absence of 1mM dATP and 4μM cytochrome C. **b.**, **d.**, **f.** Apaf-1 expression was determined in S-100 cytosol of treated and untreated BL cells by western blotting. Membranes were probed with anti β-actin for loading control of cytosolic fractions. **e.** S-100 cytosolic fraction was purified from Raji cells pre-treated with MPO (5μM) for 1hr followed by treated with etoposide (5μM) and daunorubicin (0.4μg/ml). Caspase 3 enzyme activity was then measured in the presence and absence of 1mM dATP and 4μM cytochrome c.

Apaf-1 is an essential adaptor protein required for the assembly of the apoptosome, which brings about the activation of downstream caspases, such as caspase 9 and caspase 3, and apoptosis [20]. As the apoptosome is the central execution signal downstream of mitochondria-derived death signals, its efficient assembly is imperative for an effective response against drug-induced apoptosis, which invariably involves the intrinsic death pathway. Therefore, we next asked if the resistance of primary BL cells was a function of the lack of cytosolic Apaf-1 expression. To address this, we first made use of our data indicating the ability of the small molecule experimental compounds to induce apoptosis in primary BL cells. Firstly, S-100 cytosol from primary BL cells treated for 1h with MPO (5μM), MPO-Zn (200μM), LY30 (25μM) and NaN_3_ (5μg/ml) resulted in a significant increase in cell-free caspase 3 activity, indicating efficient apoptosis induction (Figure 2a). Furthermore, while Apaf-1 expression was localized to the S100 pellets of primary BL cells, exposure of the cells to the novel small molecule compounds resulted in the redistribution of Apaf-1 to the S100 cytosols (Figure 2b). Of note, caspase 3 activity was also induced in S100 cytosol following treatment of the cells with the cholesterol lowering compound methyl beta-cyclodextrin (MCD; 200μM), also known for its ability to disrupt membrane lipid raft structures (Figure 2a). To provide conclusive evidence that the cell free system did, indeed, work as expected, we performed additional experiments using the same set of conditions with Raji cells. Results clearly showed minimal caspase 3 activity in the absence of dATP and its robust induction upon addition of dATP (Figure 2c). This was further corroborated by the appearance of Apaf-1 in the cytsolic fractions (Figure 2d). Furthermore, we performed an additional experiment using one of the compounds (MPO) that triggered the relocalization of Apaf-1 to the cytosol in combination with known inducers of apoptosis, etoposide and daunorubicin. Of note, while single agent treatment with etoposide or daunorubicin failed to induce caspase 3 activity in the cell-free model, the presence of MPO significantly enhanced sensitivity to both the agents (300% increase in caspase 3 activity as well as increased Apaf-1 release in cytosol (Figure 2e, 2f). These data provide strong evidence that the small molecule compounds facilitate the release of Apaf-1 with the resultant increase in caspase 3 activity.

### Aberrant localization of Apaf-1 in the lipid raft fractions of primary BL cells

**Figure 3:**
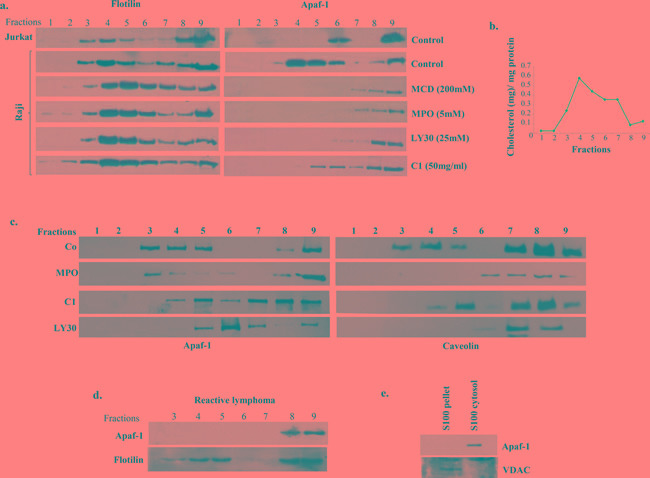
Apaf-1 is sequestered in the lipid raft fractions of Raji cells and primary BL cells **a.** Lipid raft fractions were prepared from Jurkat and Raji cells as described in Material and Methods. Raji cells were treated with MCD (200μM), MPO (5μM), C1 (50μg/ml) and LY30 (25μM) for 1hr before processing for lipid raft fractions. Apaf-1 expression was assessed in the different raft fractions by western blotting. Flotillin was used as a positive control for lipid raft fractions. **b.** Cholesterol content was determined in each lipid raft fraction according to manufacturer's instructions as described in Materials and Methods. **c.** Lipid raft fractions were prepared from primary cells derived from B-cell lymphoma and reactive lymphadenitis, as described in Material and Methods. Primary BL cells were treated with MPO (5μM), C1 (50μg/ml), or LY30 (25μM) for 1hr before processing for lipid raft fractions. Apaf-1 expression was checked in all the raft fractions by Western blotting, Caveolin was used as a positive control for lipid raft fractions. **d.** For reactive lymphadenitis, Apaf-1 was assessed in all raft fractions by western blotting. Flotillin was used as a positive control for lipid raft fractions. **e.** S-100 cytosol and pellets were prepared from primary cells from reactive lymphadenitis and Apaf-1 expression was detected by Western blotting.

Based on the findings that small molecule-induced sensitization of primary BL cells was linked to the release of Apaf-1 from the membrane fractions to the S100 fractions and our previous study linking apoptosis resistance in B cell lymphoma cell lines to membrane sequestration of Apaf-1 [14], we next assessed the role of the membrane lipid rafts in the resistance of primary BL cells to drug-induced apoptosis. Lipid raft are the cholesterol containing domains within the plasma membrane [21], and the data presented above with the cholesterol lowering compound, MCD, pointed to the possible involvement of the lipid raft domains in the mislocalization of Apaf-1 in primary BL cells. Therefore, membrane fractions were obtained using density gradient centrifugation and subjected to SDS-PAGE analysis. Interestingly, in the Raji B cell line, Apaf-1 was mostly co-localized with flotillin, which served as the positive control for lipid raft residing proteins (Figure 3a). This was distinctly different from the Jurkat T cell line, where Apaf-1 appeared in the higher cytosolic fractions. Furthermore, treatment with MCD, the lipid raft dissociating agent, resulted in the disruption of the lipid rafts and movement of Apaf-1 to the non-lipid raft fractions of the plasma membrane (Figure 3a). Similar to the effect of MCD, results also provide evidence that the sensitization to apoptosis upon exposure to the small molecule experimental compounds (MPO, MPO-Zn, C1 and LY30) was also linked to the ability of these compounds to disrupt Apaf-1 from the membrane lipid raft fractions to facilitate efficient assembly and activation of the apoptosome.

**Figure 4:**
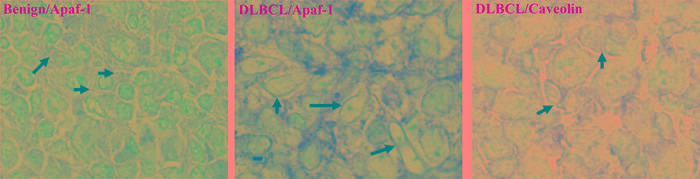
Co-localization of Apaf-1 and caveolin (lipid raft marker) in B-cell Lymphoma Apaf-1 distribution is shown in both benign and DLBCL by immunohistochemistry. Benign and DLBCL section slides were de-paraffinized and stained with anti-Apaf-1-HRP or anti-Caveolin-HRP, followed by secondary anti-HRP.

Next we assessed whether the mislocalization observed in B cell line Raji was, indeed, a mechanism operative in primary BL cells obtained from patient-derived tissue biopsies. Indeed, results obtained on primary BL cells from 10 patient biopsies revealed that Apaf-1 was sequestered in the lipid raft fractions and that treatment of the cells with the small molecule experimental compounds MPO (5μM), LY30 (25μM), C1 (50μg/ml) for 1hr, resulted in a significant decrease in the localization of Apaf-1 to the lipid raft fractions (Figure 3c, Supplementary Figure 2). To verify that sequestration of Apaf-1 was specifically limited to B-cell lymphomas, biopsies from patients with non-cancerous reactive lymphadenopathy were used. Notably, Apaf-1 was not associated with the lipid raft fractions (Figure 3d) but expressed in the S100 cytosol fractions (Figure 3e) in cells derived from non-cancerous lesions. Furthermore, we determined the cholesterol content of the fractions to validate the identity of the fractions, given the observations that these sub-domains are enriched in sphingolipids and cholesterol [22]. Indeed, fractions 3, 4 and 5 that are indicative of lipid raft fractions did show the highest level of cholesterol (Figure 3b). To further validate these findings, the same patient material was subjected to immunohistochemistry for the expression of Apaf-1. Indeed, Apaf-1 co-localized with the lipid raft marker protein caveolin (Figure 4), thereby corroborating the data presented in the preceding sections. Taken together, Apaf-1 mislocalization to lipid raft domains is a critical factor in apoptosis resistance in clinical B-cell lymphomas and strategies to disrupt these cholesterol-rich domains appear to restore death sensitivity.

### Small molecule induced re-localization of Apaf-1 to the cytosol is ROS dependent

**Figure 5:**
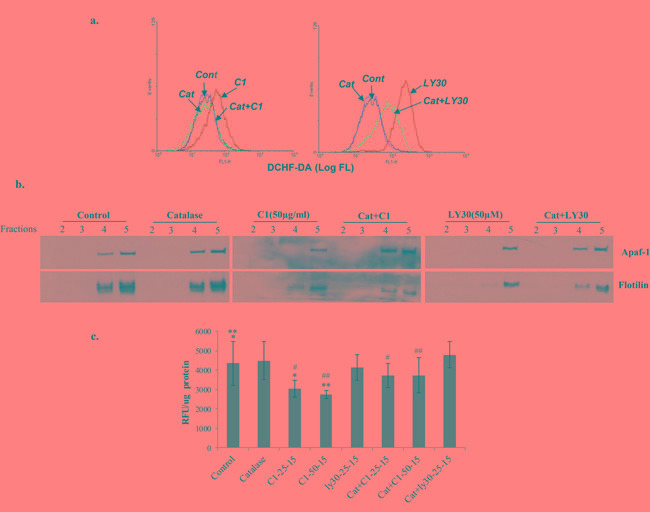
Small molecule-induced re-distribution of Apaf-1 to the cytosol is ROS dependent **a.** Raji cells were pre-incubated with Catalase (3000U/ml) followed by incubation with C1 (50μg/ml) or LY30 (25μM) for 1hr. Intracellular ROS was assessed using the fluorescent probe DCHF-DA as described in Materials and Methods. **b.** Lipid raft fractions were purified from the same cells and Apaf-1 expression was checked in fractions 2, 3, 4 and 5 by Western blotting. Flotillin was used as a control for lipid raft resident protein. **c.** Raji cells were pre-incubated with catalase (3000U/ml) followed by exposure to C1 (25, 50μg/ml) or LY30 (25, 50μM) for 15, 30 and 60 min. Lipid peroxidation was assessed using the fluorescence based assay using Cis-perinaric acid as a probe. *, **, #, ## *p* < 0.05

We were intrigued by the ability of the small molecule experimental compounds to not only dislodge Apaf-1 from the membrane lipid raft-like structures, but also to restore the sensitivity to apoptosis. Two of the molecules (LY30 and C1) have been previously reported by our group to target tumor cells via mechanisms involving an increase in intracellular ROS [15-17, 23]. As ROS have been linked to membrane lipid peroxidation and disruption of lipid rafts, we asked if the ability of C1 and LY30 to dislodge Apaf-1 from the lipid rafts was a function of intracellular ROS production. Indeed, exposure of Raji B cells to C1 (50μg/ml) or LY30 (25μM) resulted in a significant increase in intracellular ROS production measured by the change in DCF oxidation (Figure 5a). Furthermore, pre-incubation with 3000 Units of catalase significantly attenuated the increase in fluorescence, thereby indicating the involvement of hydrogen peroxide (H_2_O_2_). More importantly, both C1 and LY30 induced a change in the localization of Apaf-1 in the membrane fractions (fraction 5 as compared to fractions 4 and 5 that co-localize with flotillin), which was reversed upon *a priori* treatment with catalase (Figure 5b), further supporting the involvement of H_2_O_2_. Interestingly, not only has H_2_O_2 _been directly implicated in lipid peroxidation [24], but also ROS-induced lipid peroxidation has been linked to the modification of membrane lipid rafts [25]. Indeed, we provide evidence that treatment with the ROS producing compound C1 (25 and 50μg/ml for 15-60 minutes) resulted in an increase in membrane lipid peroxidation, which was prevented by the H_2_O_2_ scavenger catalase (Figure 5c). Taken together, these data indicate that ROS generating agents could serve as an excellent tool for the re-localization of Apaf-1 to the cytosol in B cell lymphomas that are rendered refractory to apoptosis due to the membrane sequestration of this essential adaptor protein

**Figure 6:**
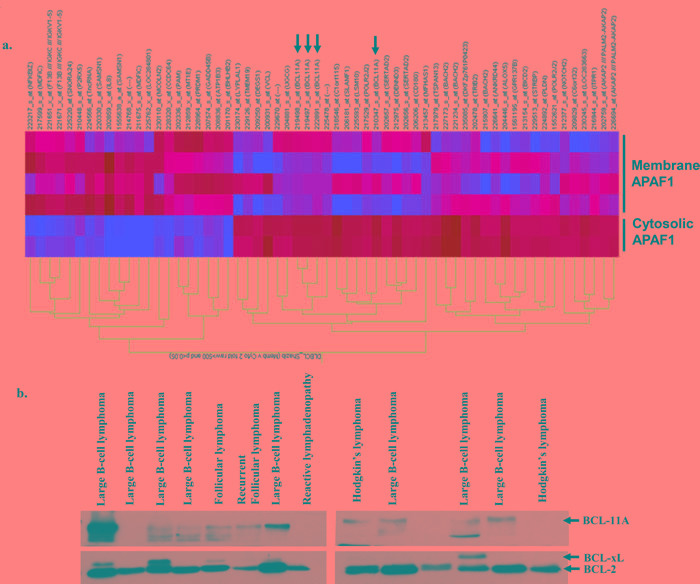
Higher expression of BCL11A in B-cell Lymphoma **a.** Gene expression profiling and analysis was done using Affymetrix HG-U133 Plus 2 microarray chips. Differentially expressed genes were selected based on mean expression intensity of 500, and > 2 fold difference in expression between samples with membrane APAF1 location *versus* samples with Cytosolic APAF1 location. **b.** Total lysates of primary cells from the same lymphoma tissues were prepared and the expression of BCL11A was assessed by western blotting.

Next, we undertook expression profiling to identify specific gene signatures and/or gene marker(s) differentially expressed in lymphomas with membrane localized Apaf-1. To check gene expression, RNA was extracted from same patients' biopsies and analyses were performed. Briefly, 186 probe sets were differentially expression by 2 fold between samples with membrane APAF1 and those with cytosolic APAF1. 132 probe sets were over-expressed and 64 under-expressed. Only probe sets of coding genes and those that are differentially expressed, also at a significance of p<0.05, are represented on the heat map (Figure 6a). Interestingly, BCL11A was amongst the genes over-expressed in the samples with membrane APAF1. The expression of BCL11A was further validated at the protein level by western blot in the same patient's biopsy material (Figure 6b). These data, albeit with a small number of samples, point to a possible correlation between membrane localization of Apaf-1 and BCL11A overexpression [26, 27], which could serve as novel indicator of drug resistance or poor prognosis in B cell lymphomas.

## DISCUSSION

DLBCL is the most common type of B-cell lymphoma that exhibits drug resistant phenotype and a high incidence of relapse [1]. As most chemotherapeutic agents preferentially activate mitochondria-driven death execution via mechanisms that trigger the induction of MOMP [2], the major focus in terms of the acquisition of drug resistance in the clinical setting has been on the anti-apoptotic members of the Bcl-2 family, such as Bcl-2 and Bcl-xL [28]. However, there is also accumulating evidence that a defect in post-MOMP signaling stalls death execution and contributes to the development of the resistant phenotype [3, 29]. In this regard, we previously reported a novel mechanism for the defective apoptosis signaling in the human B cell line, Raji, whereby defective apoptosome formation due to the absence of cytosolic Apaf-1 was identified as a critical factor [14]. In the present study, we provide clinical evidence that mislocalization of Apaf-1 may represent an important prognostic factor in the therapeutic management of DLBCL.

Apaf-1 is an essential adaptor protein, which serves as the scaffold for the recruitment of cytochrome C, pro-caspase 9, and dATP, to bring about the assembly of the apoptosome, and activation of downstream caspase 9 activation[4]. In healthy cells, Apaf-1 exists in the cytosol [30] as a monomer in a complex with dATP/ATP, a conformation that prevents its oligomerization and downstream recruitment of pro-caspase 9. Interestingly, loss of Apaf-1 has been reported in a variety of human cancers, such as melanoma, leukemia, ovarian carcinoma, bladder carcinoma and glioblastoma [8, 10, 13, 31]. Similarly, the development of resistance to death receptor- and drug-induced apoptosis in colon carcinoma was associated with downregulation of Apaf-1 [32]. Our results not only corroborate the critical importance of functional Apaf-1 in DLBCL, but also provide a novel mechanism for its loss of function. While the overall expression of Apaf-1 did not change in the clinical lymphomas we examined, the sub-cellular localization was significantly altered such that the protein was sequestered in lipid raft domains of the plasma membrane instead of being expressed in the cytosol. The importance of this mislocalization in the context of apoptosis resistance (*ex vivo*) was further corroborated by results demonstrating the restoration of caspase 3 cleavage/activation upon cytosolic redistribution of Apaf-1.

Lipid rafts are cholesterol-rich structural domains within the plasma membrane, which contain diverse protein and lipid components. Interestingly, membrane rafts are actively involved in the maintenance and regulation of signal transduction, as a number of essential signaling proteins have been shown to localize to these sub-domains, such as recruitment of receptors and/or their ligands to the rafts to facilitate intracellular signaling [22]. By the same token rafts could interfere or block cell signaling by sequestering proteins away from a specific signaling network. Of note, membrane rafts have been shown to play an important role in the activation and regulation of B cell receptor-mediated signaling [33]. Furthermore, critical components of B cell receptor signaling are absent from lipid rafts in certain disease states involving B cells, such as the EBV proteins LMP-1 and LMP-2A. While LMP-1 is involved in the sustained proliferative signal downstream of B cell receptor activation [34], LMP-2A sequesters Syk and Lyn tyrosine kinases away from the B cell receptor to block signal transduction [35]. These data seem to imply that recruitment to the lipid raft domains may not be exclusively restricted to membrane proteins or proteins with lipid-anchoring moieties. Such appears to be the case with Apaf-1, a predominantly cytosolic protein, but shown here to be sequestered in lipid raft fractions of DLBCL cells, thereby creating a non-permissive environment for apoptosome assembly and activation. The critical importance of this mislocalization of Apaf-1 in the overall biology of DLBCL is further supported by our results demonstrating the ability of small molecules that disrupt raft-like domains via their effect on cholesterol (MCD) or lipid peroxidation via ROS production (Ly30 and C1); however, the precise mechanism underlying the lipid raft recruitment of Apaf-1 is still the focus of our ongoing investigations. One could conjecture the possibility of an interaction of Apaf-1 with lipid raft-associated proteins, such as the receptor tyrosine kinase, c-Met, which is used as a prognostic marker for DLBCL [36]. Moreover, as EBV infection is a frequent finding in patients with B cell lymphoma [37], the possibility of viral proteins, such as LMP1, hijacking Apaf-1 to the lipid fractions cannot be completely ruled out. Similarly, the existence of an interaction between the WD40 domain within Apaf-1 [38] and membrane associated cytoskeleton protein(s) could be another plausible scenario. Furthermore, gene expression data also point to an interesting association between membrane-anchored Apaf-1 and overexpression of the Kruppel zinc finger gene, BCL11A. It should be pointed out that overexpression of BCL11A has previously been associated with hematopoietic malignancies, either through its gene amplification or chromosomal translocation [26, 27]. The gene expression data were confirmed at the protein level; however, these observations need to be validated in a larger cohort of patients to gain further insight into the functional relevance of the association between membrane localized Apaf-1 and BCL11A expression. Nevertheless, the preliminary association lends itself to suggest a possible marker of drug resistance and/or poor prognosis in B cell lymphomas.

One other attractive model proposed earlier by Kagan *et al.* surmised that the significant differences in the membrane composition as well as the levels of ROS in B-cell lymphomas, compared to their T cell counterparts, may affect the protein and cholesterol trafficking in the cells [39]. Although, we do not have experimental evidence to suggest differences in the membrane composition between primary cells derived from B and T cell lymphomas, results presented with small molecule experimental compounds appear to support these observations. Two small molecule experimental agents, C1 [16, 17] and LY30 [15, 23], as well as the mitochondria electron transport inhibitor NaN_3_ that trigger intracellular ROS production had a profound influence on cell-free caspase 3 activity as a result of the re-localization of Apaf-1 to the cytosol. To that end, redox-dependent alterations of lipid moieties could disrupt lipid raft structures thereby facilitating the release of sequestered Apaf-1. This is supported, at least in part, by the inhibitory effect of scavenging intracellular H_2_O_2_ with catalase. Collectively, these data suggest a novel mechanism for reinforcing apoptososme activation in DLBCL via drug-induced cytosolic redistribution of the apoptosome scaffold protein, Apaf-1, which is trapped in membrane raft sub-domains in refractory DLBCL.

## CONCLUDING REMARKS

Primary cells derived from patients with DLBCL show membrane raft sequestration of the apoptosome adaptor protein, Apaf-1. As Apaf-1 expression is critical for post-mitochondrial death execution, these data provide a novel mechanism of the refractory and aggressive nature of this sub-class of B cell lymphomas. Recent efforts have been geared at identifying novel agents that facilitate the robust assembly of apoptosome components and the major focus has been on compounds that promote the egress of cytochrome C, an essential apoptosome protein. Our data provide a novel strategy whereby the redistribution of Apaf-1 to the cytosol via drugs that act on lipid raft sub-domains through distinct mechanisms, including alterations in intracellular redox milieu, could create a favorable environment for apoptosome activation and death execution with potential therapeutic implications for the management of refractory DLBCL.

## MATERIALS AND METHODS

### Primary lymphoma cells and cancer cell lines

Tissue biopsies from lymphoma patients were obtained from National University Hospital (NUHS) of Singapore after obtaining proper informed consent from the patients. Tissues were minced and strained using MACS separation filter (Milteny Biotec, Bergisch Gladbach, Germany) to obtain a single-cell suspension. Few samples were sorted out using CD22 and CD8 antibodies by magnetic cell sorting according to manufacturer's protocol (Milteney Biotec, Bergisch Gladbach, Germany). Mononuclear cells and/or lymphocytes' enrichment was performed by Ficoll Hypaque (Sigma, St Louis, MO) gradient centrifugation. A total of 58 tissue biopsies were obtained from consenting patients (37 B cell lymphomas, 9 T cell lymphomas and 12 benign tumors) and used in this study. For *in vitro* validation, Raji (Burkitt's lymphoma) and Jurkat (T-cell Lymphoma) human cell lines were cultured in RPMI1640 supplemented with 10% FBS, 1% L-glutamine, 1% penicillin and streptomycin in a humidified incubator with 5% CO_2_.

### Determination of cell viability

Cell viability was determined by the MTT assay after exposure of 2×10^5^ primary lymphocytes/200μl/well in 96 well plates to etoposide (5μM), daunorubicin (0.4μg/ml), vincristine (5μM) (Sigma, St Louis, MO), mercaptopyridine oxide (MPO; 2.5μM), MPO-Zn (200nM), C1 (50μg/ml), LY303511 (Calbiochem, Billerica, MA) (LY30; 50μM) and TRAIL (100ng/ml) for 24 hrs. MTT assay was performed as described previously [40]. To evaluate the ability of the small molecule compounds to sensitize to death receptor-induced apoptosis, 2×10^5^ primary lymphocytes/200μl/well in 96 well plates were pretreated for 1hr with MPO (0.625μM), MPO-Zn (50nM), C1 (25μg/ml) or LY30 (25μM) prior to the addition of TRAIL (50ng/ml) for 24hrs and cell viability was determined by the MTT assay.

### Assessment of cell-free caspase-3 activity

For S-100 cytosolic extraction, 30×10^6^ cells (primary lymphocytes, Raji or Jurkat) were incubated with ice-cold S-100 buffer (20mM HEPES-KOH at pH 7.5, 10mM KCL, 1.5mM MgCl_2_, 1mM EDTA, 1mM EGTA, 1mM DTT supplemented with protease inhibitors and phosphatase inhibitors (Sigma, St Louis, MO). Cells were disrupted using a Dounce homogenizer and the pellet was centrifuged at 1000g for 10 minutes at 4^0^C. Supernatants were further centrifuged at 100,000g for 1 hr, and the resulting supernatant (S-100 cytosol) was stored at -80^o^C or used immediately. The S-100 pellet was dissolved in 1X RIPA lysis buffer with protease and phosphatase inhibitors and stored at -80^0^C or used immediately. For cell free caspase-3 activation, S-100 cytosol (120μg) from cells treated with MPO (5μM), MPO-Zn (200μM), LY30 (25μM), NaN_3_ (5μg/ml), etoposide (5μΜ) or daundorubicin (0.5μl/μl) were incubated in the presence or absence of 1mM dATP and 4μM cytochrome C (Sigma, St Louis, MO) for 30min at 37^0^C. Caspase-3 activity was then measured by using fluorogenic substrate DEVD-AFC as described previously [40].

### Western blot analysis

S-100 cytosol and pellet fractions or lipid raft fractions were denatured in loading buffer and resolved on 10% sodium dodecyl sulfate-polyacrylamide gel electrophoresis (SDS-PAGE) followed by transfer to PVDF membranes. Immunoblotting was performed with antibodies against Apaf-1 (Chemicon, CA), β-actin, Bcl-2, Bcl-xl, VDAC-1 (Santa-Cruz, Dallas, TX), BCL11A, caveolin and flotillin (Abcam, Cambridge, UK) and then probed with the isotype specific secondary IgG-HRP as required. Membranes were then exposed to Super Signal Substrate Western Blotting Kit (Pierce, Rockford, IL, USA).

### Purification of membrane lipid raft fraction

For lipid raft extraction, 40×10^6^ cells (primary lymphocytes, Raji or Jurkat) were incubated in ice-cold HEPES buffer (pH 7.4) for 20 minutes. Cells were disrupted using a Dounce homogenizer followed by sonication and the lysates were centrifuged at 1000g for 10 minutes at 4^o^C. The resulting supernatant was loaded onto a gradient of 80%, 30%, and 5% sucrose and centrifuged at 38000 RPM for 21 hours at 4^o^C. 500ul of the fraction was collected from the top and stored at -80^0^ or used immediately. Fractions were used for the determination of cholesterol content using the cholesterol quantitation kit (Calbiochem, Billerica, MA) following the manufacturers recommended protocol, as well as subjected to SDS-PAGE for western blot analysis of membrane proteins.

### Determination of intracellular ROS and lipid peroxidation

2×10^6^ primary lymphocytes or Raji cells were treated with or without C1 (25 and 50μg/ml) or LY30 (25 and 50μM) for 15, 30 and 60minutes. Cells were incubated with catalase (3000U/ml) one hour prior to the addition of the drugs and cellular ROS content was measured by loading cells with DCHF-DA (Molecular Probes, Eugene, OR), as described previously [23]. For the assessment of lipid peroxidation, samples were incubated with cis-perinaric acid (10μM) (Sigma, St Louis, MO) for 1hr at 37^o^C, and a change in the fluorescence emission at 418nm following excitation at 324nm was used to indicate an increase in lipid peroxidation.

### Immunohistochemistry

Formalin-fixed paraffin embedded lymphoma sections were obtained from National University Hospital Singapore, Singapore. The slides were de-paraffinized and endogenous peroxidase activity was inhibited with 3% hydrogen peroxide in 1X PBS. Sections were stained with HRP-conjugated primary antibody for overnight followed by the secondary anti-HRP antibody. DAB substrate (DAKO, Denmark) was added for 5 min at room temperature before washing slides with distilled water. Slides were counterstained with hematoxylin for 5 sec before being mounted with aqueous mounting media and viewed under a light microscope.

### Gene expression profiling and analysis

Gene expression microarray was performed using Affymetrix HG-U133 Plus 2 microarray chips. CEL files were processed using the MAS5 algorithm. Differentially expressed genes were selected based on mean expression intensity of 500, and >2 fold  difference in expression between samples with membrane APAF1 location versus samples with Cytosolic APAF1 location with t-test p-value of 0.05. 

## SUPPLEMENTARY MATERIALS FIGURES AND TABLES


