# Supplementary material for: Aberrant localization of apoptosis protease activating factor-1 in lipid raft sub-domains of diffuse large B cell lymphomas
Source: Oncotarget. 2016 Nov 14;7(51):83964–75. doi: 10.18632/oncotarget.13336 (PMC5356638; doi:10.18632/oncotarget.13336)
Supplement: Supplementary file 1 [file oncotarget-07-83964-s001.pdf]

Aberrant localization of apoptosis protease activating factor-1 in lipid raft sub-domains of diffuse large B cell lymphomas

Supplementary Material

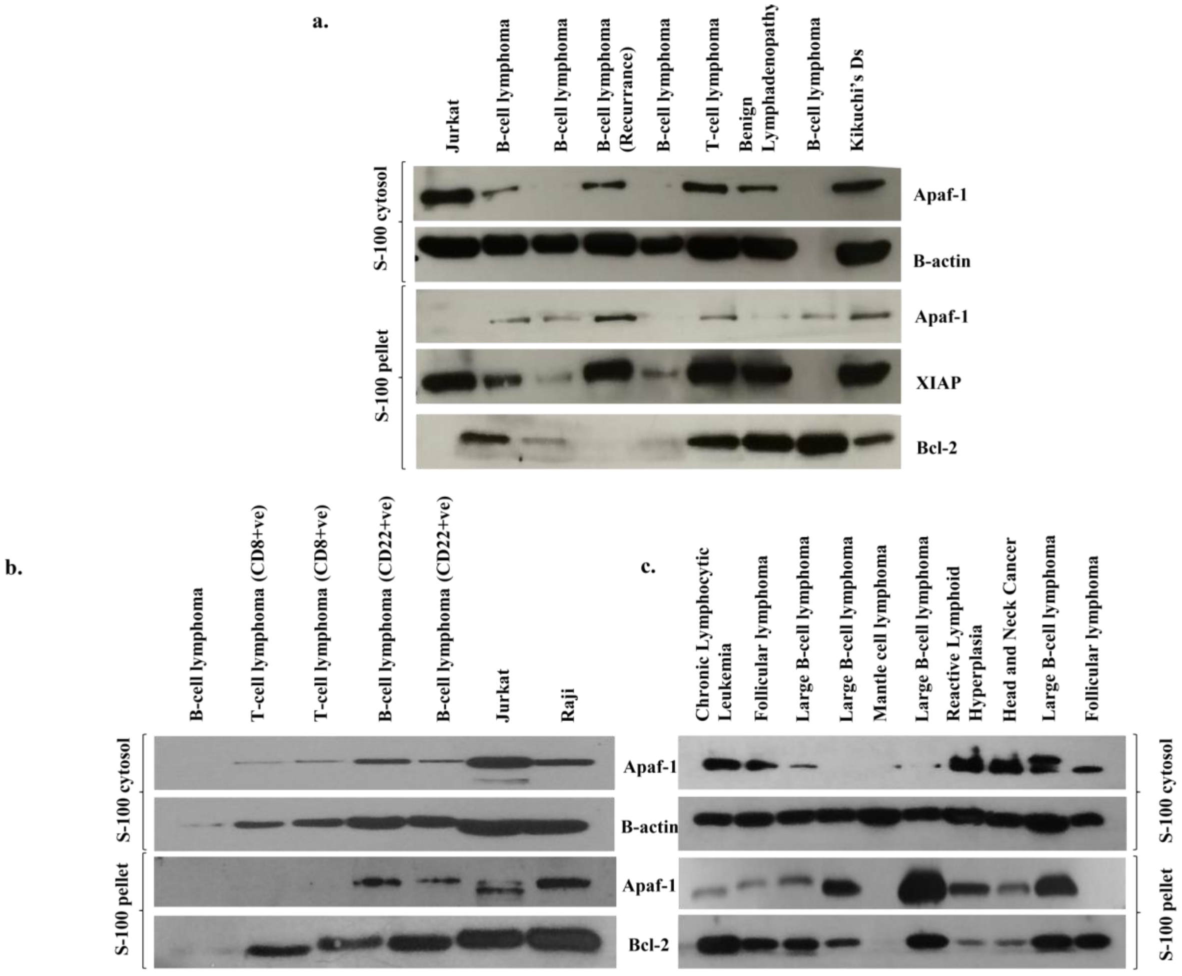

Figure S1: Lower expression of Apaf-1 in the cytoplasm of primary BL cells. (a, c) S-100 cytosolic and pellet fractions of primary cells from B-cell, T-cell lymphomas and Benign lymphoma as well as from Jurkat cell lines were resolved by SDS-PAGE, transferred to PVDF and Apaf-1 detected by immunoblotting using anti-Apaf-1 **(b)** CD22 and CD8 cells were sorted using magnetic beads from B-cell and T-cell Lymphoma respectively. S-100 cytosolic fractions were prepared and protein was detected by western blot.

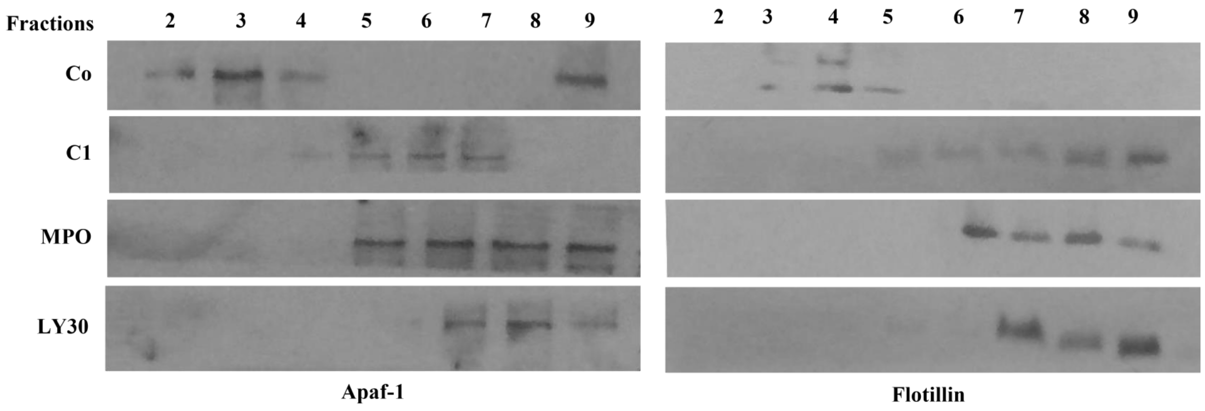

Figure S2: Apaf-1 is sequestered in the lipid raft fractions of primary BL cells.

Lipid raft fractions were prepared from primary cells derived from B-cell lymphoma as described in Material and Methods. Primary BL cells were treated with MPO (5µM), C1 (50µg/ml), or LY30 (25µM) for 1hr before processing for lipid raft fractions. Apaf-1 expression was checked in all the raft fractions by Western blotting, Flotillin was used as a positive control for lipid raft fractions
